# Supplementary material for: A Polynomial-Exponent Model for Calibrating the Frequency Response of Photoluminescence-Based Sensors
Source: Sensors (Basel). 2020 Aug 18;20(16):4635. doi: 10.3390/s20164635 (PMC7472340; doi:10.3390/s20164635)
Supplement: Supplementary file 1 [file sensors-20-04635-s001.zip › sensors-877605-supplementary materials/supplementary materials.pdf]

sensors

MDPI

Article

Supplementary Materials: A Polynomial-Exponent Model for Calibrating the Frequency Response of Photoluminescence-Based Sensors

Angel de la Torre [1], Santiago Medina-Rodríguez [2], Jose C. Segura [1], and Jorge F. Fernández-Sánchez [3,*]

1 Department Signal Theory, Networking and Communications, University of Granada, 18071 Granada, Spain; atv@ugr.es (A.d.l.T.); segura@ugr.es (J.C.S.)
2 By Techdesign S.L., Madrid, 28500 Arganda del Rey, Spain; santiago.medina@by.com.es
3 Department of Analytical Chemistry, University of Granada, 18071 Granada, Spain
* Correspondence: jffernan@ugr.es; Tel.: +34-958-240451

Received: 8 July 2020; Accepted: 13 August 2020 ; Published: date

1. Complementary Mathematical Formulation

1.1. Functions $C(\phi)$ and $\phi(C)$ for the Models PE1, PE2 and P2

This section provides mathematical expressions for $C(\phi)$ and $\phi(C)$ for the models proposed in the manuscript.

1.1.1. 1st Degree Polynomial-Exponent Model (PE1)

$$C(\phi) = a_0 + a_1\phi^{\alpha} \qquad \phi(C) = \left(\frac{C - a_0}{a_1}\right)^{1/\alpha} \tag{1}$$

1.1.2. 2nd Degree Polynomial-Exponent Model (PE2)

$$C(\phi) = a_0 + a_1\phi^{\alpha} + a_2\phi^{2\alpha} \qquad \phi(C) = \left(\frac{-a_1 + \sqrt{a_1^2 - 4a_2(a_0 - C)}}{2a_2}\right)^{1/\alpha} \tag{2}$$

1.1.3. 2nd Degree Polynomial Model (P2)

$$C(\phi) = a_0 + a_1\phi^{-1} + a_2\phi^{-2} \qquad \phi(C) = \left(\frac{-a_1 + \sqrt{a_1^2 - 4a_2(a_0 - C)}}{2a_2}\right)^{-1} \tag{3}$$

1.2. Sensitivity and Response at Null Concentration

The response at null concentration ϕ_0 and the sensitivity K are defined, respectively as:

$$\phi_0 \equiv \lim_{C\to 0} \phi(C) \qquad K \equiv \lim_{C\to 0} \frac{1}{\phi(C)} \left|\frac{\partial \phi(C)}{\partial C}\right| = \lim_{C\to 0} \frac{1}{\phi_0} \left|\frac{\partial \phi(C)}{\partial C}\right| = \lim_{C\to 0} \frac{1}{\phi_0} \left|\frac{\partial C(\phi)}{\partial \phi}\right|^{-1} \tag{4}$$

From these definitions, ϕ_0 and K can be derived for the models PE1, PE2 and P2.

1.2.1. 1st Degree Polynomial-Exponent Model (PE1)

$$C(\phi) = a_0 + a_1\phi^\alpha \qquad \phi(C) = \left(\frac{C-a_0}{a_1}\right)^{1/\alpha} \qquad \frac{\partial\phi(C)}{\partial C} = \left(\frac{\partial C(\phi)}{\partial\phi}\right)^{-1} = \frac{1}{a_1\alpha\phi^{\alpha-1}} \tag{5}$$

$$\phi_0 = \lim_{C\to 0}\left(\frac{C-a_0}{a_1}\right)^{1/\alpha} = \left(\frac{-a_0}{a_1}\right)^{1/\alpha} \qquad K = \lim_{C\to 0}\frac{1}{\phi}\left|\frac{1}{a_1\alpha\phi^{\alpha-1}}\right| = \left|\frac{1}{a_1\alpha\phi_0^\alpha}\right| = \left|\frac{1}{a_1\alpha(-a_0/a_1)}\right| = \left|\frac{1}{a_0\alpha}\right| \tag{6}$$

1.2.2. 2nd Degree Polynomial-Exponent Model (PE2)

$$C(\phi) = a_0 + a_1\phi^\alpha + a_2\phi^{2\alpha} \qquad \phi(C) = \left(\frac{-a_1+\sqrt{a_1^2-4a_2(a_0-C)}}{2a_2}\right)^{1/\alpha} \tag{7}$$

$$\frac{\partial\phi(C)}{\partial C} = \left(\frac{\partial C(\phi)}{\partial\phi}\right)^{-1} = \frac{1}{\alpha a_1\phi^{\alpha-1}+2\alpha a_2\phi^{2\alpha-1}} \tag{8}$$

$$\phi_0 = \lim_{C\to 0}\left(\frac{-a_1+\sqrt{a_1^2-4a_2(a_0-C)}}{2a_2}\right)^{1/\alpha} = \left(\frac{-a_1+\sqrt{a_1^2-4a_0a_2}}{2a_2}\right)^{1/\alpha} \tag{9}$$

$$K = \lim_{C\to 0}\frac{1}{\phi}\left|\frac{1}{\alpha a_1\phi^{\alpha-1}+2\alpha a_2\phi^{2\alpha-1}}\right| = \left|\frac{1}{\alpha a_1\phi_0^\alpha+2\alpha a_2\phi_0^{2\alpha}}\right| \tag{10}$$

1.2.3. 2nd Degree Polynomial Model (P2)

$$C(\phi) = a_0 + a_1\phi^{-1} + a_2\phi^{-2} \qquad \phi(C) = \left(\frac{-a_1+\sqrt{a_1^2-4a_2(a_0-C)}}{2a_2}\right)^{-1} \tag{11}$$

$$\frac{\partial\phi(C)}{\partial C} = \left(\frac{\partial C(\phi)}{\partial\phi}\right)^{-1} = \frac{-1}{a_1\phi^{-2}+2a_2\phi^{-3}} \tag{12}$$

$$\phi_0 = \lim_{C\to 0}\left(\frac{-a_1+\sqrt{a_1^2-4a_2(a_0-C)}}{2a_2}\right)^{-1} = \left(\frac{-a_1+\sqrt{a_1^2-4a_0a_2}}{2a_2}\right)^{-1} \tag{13}$$

$$K = \lim_{C\to 0}\frac{1}{\phi}\left|\frac{-1}{a_1\phi^{-2}+2a_2\phi^{-3}}\right| = \left|\frac{1}{a_1\phi_0^{-1}+2a_2\phi_0^{-2}}\right| \tag{14}$$

1.3. Generic Calibration Procedure

Given a calibration dataset with N calibration values $\{(\phi_1, C_1), (\phi_2, C_2), \ldots, (\phi_N, C_N)\}$ (where each calibration point is a pair (ϕ_n, C_n) describing the instrument response at a given concentration), the calibration procedure consists in the minimization of the error function $E(\Lambda, \{(\phi_1, C_1), (\phi_2, C_2), \ldots, (\phi_N, C_N)\})$ with respect to the model parameters $\Lambda = \{\lambda_1, \lambda_2, \ldots, \lambda_M\}$ (For details about the generic calibration procedure, see: S. Medina et al., "On the calibration of chemical sensors based on photoluminescence: selecting the appropriate optimization criterion",

Sensors and Actuators B: Chemical, 212, (2015) 278-286, http://dx.doi.org/10.1016/j.snb.2015.02.022 .). The error E is minimized by finding the zeros of the partials:

$$\frac{\partial E}{\partial \lambda_m} = 0 \quad \forall m = 1, \ldots, M \tag{15}$$

If we define:

$$f_m(\lambda_m) \equiv \frac{\partial E}{\partial \lambda_m} \tag{16}$$

the minimization of the error E is equivalent to finding the values λ_m that simultaneously cancel $f_m(\lambda_m)$ for each m. The Newton's method can be applied for the iterative estimation of the roots of $f_m(\lambda_m)$ (and therefore to the estimation of the model parameters minimizing E):

$$\lambda_m^{t+1} = \lambda_m^t - \frac{f_m(\lambda_m^t)}{f'_m(\lambda_m^t)} \tag{17}$$

where t is the iteration and $f'_m(\lambda_m)$ is the derivative of $f_m(\lambda_m)$ with respect to λ_m. This method provides a generic procedure for calibration where the parameter λ_m can be calculated at iteration $t+1$ from its value at iteration t. The calibration would start with an initialization of the model parameters λ_m^0. If we evaluate $E(\lambda_m^t)$, $E(\lambda_m^t + \epsilon)$ and $E(\lambda_m^t - \epsilon)$ (being ϵ an infinitesimal increment in λ_m), the following approaches are possible:

$$f_m(\lambda_m^t) \approx \frac{E(\lambda_m^t + \epsilon) - E(\lambda_m^t - \epsilon)}{2\epsilon} \qquad \left.\frac{\partial f_m(\lambda_m)}{\partial \lambda_m}\right|_{\lambda_m^t} \approx \frac{E(\lambda_m^t + \epsilon) + E(\lambda_m^t - \epsilon) - 2E(\lambda_m^t)}{\epsilon^2} \tag{18}$$

and the iterative procedure can be expressed as:

$$\lambda_m^{t+1} \approx \lambda_m^t - \frac{\epsilon}{2} \frac{E(\lambda_m^t + \epsilon) - E(\lambda_m^t - \epsilon)}{E(\lambda_m^t + \epsilon) + E(\lambda_m^t - \epsilon) - 2E(\lambda_m^t)} \tag{19}$$

Therefore, given the calibration data, the iterative procedure would be carried out as follows:

1. The model parameters are initialized: $\lambda_m^0 \quad \forall m = 1, \ldots, M$.
2. For each iteration t, until convergence:
   (a) For each parameter to optimize λ_m, errors are calculated:
      - $E(\lambda_1^t, \ldots, \lambda_m^t, \ldots, \lambda_M^t)$
      - $E(\lambda_1^t, \ldots, \lambda_m^t + \epsilon, \ldots, \lambda_M^t)$
      - $E(\lambda_1^t, \ldots, \lambda_m^t - \epsilon, \ldots, \lambda_M^t)$

   (b) λ_m^{t+1} is estimated using equation (19).
3. When convergence is reached, the calibrated model parameters are $\hat{\Lambda} = \{\hat{\lambda}_1, \hat{\lambda}_2, \ldots, \hat{\lambda}_M\}$ and the minimized calibration error would be $E(\hat{\Lambda}, \{(x_1, C_1), (x_2, C_2), \ldots, (x_N, C_N)\})$.

This generic calibration procedure has the disadvantage of being a M-dimensional search, and at each iteration all the parameters should be independently estimated. However, taking into account the model, the conditions $f_m(\lambda_m) = 0$ allow to write some of the parameters as a function of the others, which reduces the dimensionality of the search. In the next section, the calibration procedure is particularized for the PE model.

1.4. Calibration Formulas for the Polynomial-Exponent Model

In the polynomial-exponent model, the concentration as a function of the analytical signal is modeled as:

$$C(\phi) = a_0 + a_1\phi^{\alpha} + a_2\phi^{2\alpha} + \ldots + a_p\phi^{p\alpha} \tag{20}$$

and for a calibration dataset, the error to be minimized is:

$$E = \sum_n w_n (C(\phi_n) - C_n)^2 \tag{21}$$

where the weights w_n can be set to 1 if the criterion is the minimization of the error in concentration, $w_n = 1/C_n^2$ if the criterion is minimization of the relative error in concentration, or to $w_n = |\partial C(\phi)/\partial\phi|_{C_n}^{-2}$ if the criterion is the minimization of the error in the analytical signal (For details about the minimization criteria, see the previously referred article, Sensors and Actuators B: Chemical, 212, (2015) 278-286.), that is:

$$w_n = \left| \frac{\partial C(\phi)}{\partial \phi} \right|_{C_n}^{-2} = \left(a_1 \alpha \phi_n^{\alpha-1} + 2\, a_2 \alpha \phi_n^{2\alpha-1} + \ldots + p\, a_p \alpha \phi_n^{p\alpha-1} \right)^{-2} \tag{22}$$

The application of these weights provide the calibration criteria (b), (c) and (a), respectively, described in the above referred article.

The partials of the error with respect to the polynomial coefficients are:

$$\frac{\partial E}{\partial a_0} = 2 \sum_n w_n (a_0 + a_1 \phi_n^{\alpha} + a_2 \phi_n^{2\alpha} + \ldots + a_p \phi_n^{p\alpha} - C_n) \tag{23}$$

$$\frac{\partial E}{\partial a_1} = 2 \sum_n w_n (a_0 + a_1 \phi_n^{\alpha} + a_2 \phi_n^{2\alpha} + \ldots + a_p \phi_n^{p\alpha} - C_n) \phi_n^{\alpha} \tag{24}$$

$$\frac{\partial E}{\partial a_2} = 2 \sum_n w_n (a_0 + a_1 \phi_n^{\alpha} + a_2 \phi_n^{2\alpha} + \ldots + a_p \phi_n^{p\alpha} - C_n) \phi_n^{2\alpha} \tag{25}$$

$$\vdots$$

$$\frac{\partial E}{\partial a_p} = 2 \sum_n w_n (a_0 + a_1 \phi_n^{\alpha} + a_2 \phi_n^{2\alpha} + \ldots + a_p \phi_n^{p\alpha} - C_n) \phi_n^{p\alpha} \tag{26}$$

The conditions $\partial E/\partial a_i = 0$ (with $i = 0, \ldots, p$) constitute a linear equation system:

$$\frac{\partial E}{\partial a_0} = 0 \quad \Leftrightarrow \quad a_0 \sum_n w_n + a_1 \sum_n w_n \phi_n^{\alpha} + a_2 \sum_n w_n \phi_n^{2\alpha} + \ldots + a_p \sum_n w_n \phi_n^{p\alpha} = \sum_n w_n C_n \tag{27}$$

$$\frac{\partial E}{\partial a_1} = 0 \quad \Leftrightarrow \quad a_0 \sum_n w_n \phi_n^{\alpha} + a_1 \sum_n w_n \phi_n^{2\alpha} + a_2 \sum_n w_n \phi_n^{3\alpha} + \ldots + a_p \sum_n w_n \phi_n^{(p+1)\alpha} = \sum_n w_n C_n \phi_n^{\alpha} \tag{28}$$

$$\frac{\partial E}{\partial a_2} = 0 \quad \Leftrightarrow \quad a_0 \sum_n w_n \phi_n^{2\alpha} + a_1 \sum_n w_n \phi_n^{3\alpha} + a_2 \sum_n w_n \phi_n^{4\alpha} + \ldots + a_p \sum_n w_n \phi_n^{(p+2)\alpha} = \sum_n w_n C_n \phi_n^{2\alpha} \tag{29}$$

$$\vdots$$

$$\frac{\partial E}{\partial a_p} = 0 \quad \Leftrightarrow \quad a_0 \sum_n w_n \phi_n^{p\alpha} + a_1 \sum_n w_n \phi_n^{(p+1)\alpha} + a_2 \sum_n w_n \phi_n^{(p+2)\alpha} + \ldots + a_p \sum_n w_n \phi_n^{(2p)\alpha} = \sum_n w_n C_n \phi_n^{p\alpha} \tag{30}$$

This equation system can be written in matrix form as:

$$A \cdot \vec{a} = \vec{B} \tag{31}$$

where the vector $\vec{a}$ contains the polynomial coefficients $a_0, \ldots, a_p$, and A and $\vec{B}$ are, respectively, a square matrix and a column vector whose components are:

$$A_{i,j} = \sum_n w_n \phi_n^{(i+j)\alpha} \qquad B_i = \sum_n w_n C_n \phi_n^{i\alpha} \qquad i, j = 0, \ldots, p \tag{32}$$

and therefore, given α, the polynomial coefficients are directly obtained by inverting the matrix A as:

$$\boxed{\vec{a} = A^{-1} \cdot \vec{B}} \tag{33}$$

2. Octave/MatLab Implementation of the PE1, PE2 and P2 Calibration Procedures

This section provides Octav/MatLab functions for the calibration of the PE1, PE2 and P2 models. The syntax and implementation of these procedures is similar to that for the Stern-Volmer, Lehrer and Demas models (including the proposed minimization criteria) previously published in the above referred article (Sensors and Actuators B: Chemical, 212, (2015) 278-286.). In addition to the code providing the calibration, an Octave/Matlab script using these functions is also included as an example.

2.1. Function for Fitting the 2nd Degree Polynomial Model: Model_P2.m

```
%%%%%%%%%%%%%%%%%%%%%%%%%%%%%%%%%%%%%%%%%%%%%%%%%%%%%%%%%%%%%%%%%%%%%%%%%%
% function [a0,a1,a2,PHI_0,K,R2,Erms] = model_P2(phi_n,C_n,NITER,CRITERION,DIAGRAM,REPORT)
%%%%%%%%%%%%%%%%%%%%%%%%%%%%%%%%%%%%%%%%%%%%%%%%%%%%%%%%%%%%%%%%%%%%%%%%%%%%%%%%%%%%%
%%% Developed by A. de la Torre, S. Medina-Rodriguez, JC. Segura and
%%%   J.F. Fernandez-Sanchez
%%%   (atv@ugr.es smedina@ugr.es, segura@ugr.es, jffernan@ugr.es)
%%% Dpt. Signal Theory Networking and Communications / Dpt. Analytical Chemistry
%%% University of Granada, May 2020
%%%%%%%%%%%%%%%%%%%%%%%%%%%%%%%%%%%%%%%%%%%%%%%%%%%%%%%%%%%%%%%%%%%%%%%%%%%%%%%%%%%%%%
%   This function provides an estimation of the parameters of the
% 2nd order Polynomic model (P2) given observations of
% photoluminiscece consisting on analytical signal and concentration (phi_n, C_n)
%   The variable NITER indicates the number of iterations
%   The variable CRITERION defines the calibration criterion ('A' 'B' 'C')
%   The variables DIAGRAM and REPORT provides some information and plots
% while the model parameters are estimated
%   The output parameters are:
%        a0,a1,a2   The polynomial coefficients
%        PHI_0      The response of the model at null concentration
%        K          The sensitivity of the model
%        R2         Determination coefficient
%        Erms       RMS error for calibration data
%   PE2 model:
%        C = a0 + a1 / phi + a2 / phi^2
%
%   Procedure for optimization:
%      it is a direct estimation for criteria (b) and (c);
%      it is an iterative procedure for criterion (a)
%
%    Example:
%         C_n=[0.01 0.03 0.1 0.3 1];
%         phi_n=[35 30 25 20 15];
%         [a0,a1,a2,PHI_0,K,R2,Erms] = model_P2(phi_n,C_n,100,'c',1,1);
%%%%%%%%%%%%%%%%%%%%%%%%%%%%%%%%%%%%%%%%%%%%%%%%%%%%%%%%%%%%%%%%%%%%%%%%%%
function [a0,a1,a2,PHI_0,K,R2,Erms] = model_P2(phi_n,C_n,NITER,CRITERION,DIAGRAM,REPORT)
N=length(C_n);
if N~=length(phi_n)
    fprintf('Error: phi_n and C_n should have the same length\n');
    a0=[]; a1=[]; a2=[]; K=[]; PHI_0=[]; R2=[]; Erms=[]; return;
end

Report_iterations = 0;
switch lower(CRITERION)
    case 'b'
        w_n = ones(size(C_n));
        [a0,a1,a2,R2,Erms]=P2_estimation_bc(phi_n,C_n,w_n,'b');
    case 'c'
        cond = C_n>0;
        if sum(cond)~=length(phi_n)
            fprintf('...Warning!: null concentration ignored for (c) criterion.\n');
            phi_n=phi_n(cond); C_n=C_n(cond);
        end
        w_n = 1./(C_n.*C_n);
        [a0,a1,a2,R2,Erms]=P2_estimation_bc(phi_n,C_n,w_n,'c');
    case 'a'
        [a0,a1,a2,R2,Erms]=P2_estimation_a(phi_n,C_n,NITER,Report_iterations);
    otherwise
```

```
        fprintf('Error: CRITERION should be ''a'' ''A'' ''b'' ''B'' ''c'' or ''C''\n');
        a0=[]; a1=[]; a2=[]; K=[]; PHI_0=[]; R2=[]; Erms=[]; return;
end

PHI_0 = ((-a1 + sqrt(a1*a1-4*a0*a2))/(2*a2))^(-1);
K = abs(1/(a1*PHI_0^(-1)+2*a2*PHI_0^(-2)));
if DIAGRAM
    figure(DIAGRAM)
    D_phi=(max(phi_n)-min(phi_n))/200;
    x_grafica = min(phi_n):D_phi:max(phi_n);
    y=a0+a1*x_grafica.^(-1)+a2*x_grafica.^(-2);
    plot(x_grafica,y,'-r',phi_n,C_n,'ok')
    xlabel('analytical signal')
    ylabel('concentration (%)')
end
if REPORT
    fprintf('Pol-2(%s):   E=%.4f  R2=%.4f  a0=%.4f  a1=%.4f  a2=%.4f  PHI_0=%.4f  K=%.4f\n',...
        CRITERION,Erms,R2,a0,a1,a2,PHI_0,K);
end
return;
%%%%%%%%%%%%%%%%%%%%%%%%%%%%%%%%%%%%%%%%%%%%%%%%%%%%%%%%%%%%%%%%%%%%%%%%%%%%
function [a0,a1,a2,R2,Erms]=P2_estimation_a(phi_n,C_n,NITER,REPORT)
% pre-scaling
phi_scale=max(phi_n);
phi_n=phi_n/phi_scale;
% iterative estimation for criterion (a)
% first step: initalization with P2(c)
w_a=zeros(size(C_n));
cond=C_n>0;
w_a(cond)=1./(C_n(cond).^2);
[a0,a1,a2]=P2_estimation_bc(phi_n,C_n,w_a,'c');
% reestimation of weights and new iterations:
for iter=1:NITER
    w_a=(a1./(phi_n.^(2))+2*a2./(phi_n.^(3))).^(-2);
    [a0,a1,a2,R2,Erms]=P2_estimation_bc(phi_n,C_n,w_a,'c');
    if REPORT, fprintf('Iter:%d  E:%f  a0:%f  a1:%f  a2=%f\n',iter,Erms,a0,a1,a2); end;
end
% de-scaling
phi_n=phi_n*phi_scale;
a1=a1*phi_scale;
a2=a2*phi_scale*phi_scale;
% statistical results
phi_estim = ((-a1+sqrt(a1*a1-4*a2*(a0-C_n)))/(2*a2)).^(-1);
SS_res = mean((phi_estim - phi_n).^2);
Erms = sqrt(SS_res);
mu = mean(phi_n);
SS_tot = mean((phi_n - mu).^2);
R2 = 1 - SS_res/SS_tot;
return;
%%%%%%%%%%%%%%%%%%%%%%%%%%%%%%%%%%%%%%%%%%%%%%%%%%%%%%%%%%%%%%%%%%%%%%%%%%%%
function [a0,a1,a2,R2,Erms]=P2_estimation_bc(phi_n,C_n,w_n,CRITERION)
% pre-scaling
phi_scale=max(phi_n);
phi_n=phi_n/phi_scale;
alpha=-1;
[E0,a0,a1,a2] = Error_estimation_PE2_bc(phi_n,C_n,w_n,alpha);
% de-scaling
phi_n=phi_n*phi_scale;
a1=a1/phi_scale^alpha;
a2=a2/phi_scale^(2*alpha);
% statistical results
C_estim=a0+a1*phi_n.^alpha+a2*phi_n.^(2*alpha);
SS_res = mean(w_n.*(C_estim - C_n).^2);
Erms = sqrt(SS_res);
mu = mean(C_n);
SS_tot = mean(w_n.*(C_n - mu).^2);
R2 = 1 - SS_res/SS_tot;
if CRITERION == 'c'
    R2=1-SS_res;
end
return;
%%%%%%%%%%%%%%%%%%%%%%%%%%%%%%%%%%%%%%%%%%%%%%%%%%%%%%%%%%%%%%%%%%%%%%%%%%%%
function [E,a0,a1,a2] = Error_estimation_PE2_bc(phi_n,C_n,w_n,alpha)
% definitions
f=phi_n.^alpha;
```

```
X=ones(size(C_n));
Xf=X.*f; Xff=Xf.*f; Xfff=Xff.*f; Xffff=Xfff.*f;
Xc=C_n; Xcf=Xc.*f; Xcff=Xcf.*f;
% elements of matrices
A0=sum(w_n.*X); A1=sum(w_n.*Xf); A2=sum(w_n.*Xff);
A3=sum(w_n.*Xfff); A4=sum(w_n.*Xffff);
B0=sum(w_n.*Xc); B1=sum(w_n.*Xcf); B2=sum(w_n.*Xcff);
% matrices
A=[A0 A1 A2; A1 A2 A3; A2 A3 A4];
B=[B0; B1; B2];
% coeficients a0 a1 a2:
x=A\B; a0=x(1); a1=x(2); a2=x(3);
% error estimation
C_estim = a0 + a1*f + a2*f.*f;
E=mean(w_n.*(C_estim-C_n).^2);
return;
%%%%%%%%%%%%%%%%%%%%%%%%%%%%%%%%%%%%%%%%%%%%%%%%%%%%%%%%%%%%%%%%%%%%%%%%%%%
```

2.2. Function for Fitting the 1st Degree Polynomial-Exponent Model: Model_PE1.m

```
%%%%%%%%%%%%%%%%%%%%%%%%%%%%%%%%%%%%%%%%%%%%%%%%%%%%%%%%%%%%%%%%%%%%%%%%%%%
% function [a0,a1,alpha,PHI_0,K,R2,Erms] = model_PE1(phi_n,C_n,NITER,CRITERION,DIAGRAM,REPORT)
%%%%%%%%%%%%%%%%%%%%%%%%%%%%%%%%%%%%%%%%%%%%%%%%%%%%%%%%%%%%%%%%%%%%%%%%%%%%%%%%%%%%%%
%%% Developed by A. de la Torre, S. Medina-Rodriguez, JC. Segura and
%%%   J.F. Fernandez-Sanchez
%%%   (atv@ugr.es smedina@ugr.es, segura@ugr.es, jffernan@ugr.es)
%%% Dpt. Signal Theory Networking and Communications / Dpt. Analytical Chemistry
%%% University of Granada, May 2020
%%%%%%%%%%%%%%%%%%%%%%%%%%%%%%%%%%%%%%%%%%%%%%%%%%%%%%%%%%%%%%%%%%%%%%%%%%%%%%%%%%%%%%
%   This function provides an estimation of the parameters of the
% 1st order Polynomic-Exponent model (PE1) given observations of
% photoluminiscece consisting on analytical signal and concentration (phi_n, C_n)
%   The variable NITER indicates the number of iterations
%   The variable CRITERION defines the calibration criterion ('A' 'B' 'C')
%   The variables DIAGRAM and REPORT provides some information and plots
% while the model parameters are estimated
%   The output parameters are:
%        a0,a1      The polynomial coefficients
%        alpha      The exponent
%        PHI_0      The response of the model at null concentration
%        K          The sensitivity of the model
%        R2         Determination coefficient
%        Erms       RMS error for calibration data
%   PE2 model:
%        C = a0 + a1 phi^alpha
%
%   Procedure for optimization:
%      it is a 1-D search implemented with Newton's method
%
%    Example:
%         C_n=[0.01 0.03 0.1 0.3 1];
%         phi_n=[35 30 25 20 15];
%         [a0,a1,alpha,PHI_0,K,R2,Erms] = model_PE1(phi_n,C_n,100,'c',1,1);
%%%%%%%%%%%%%%%%%%%%%%%%%%%%%%%%%%%%%%%%%%%%%%%%%%%%%%%%%%%%%%%%%%%%%%%%%%%
function [a0,a1,alpha,PHI_0,K,R2,Erms] = model_PE1(phi_n,C_n,NITER,CRITERION,DIAGRAM,REPORT)
N=length(C_n);
if N~=length(phi_n)
    fprintf('Error: phi_n and C_n should have the same length\n');
    a0=[]; a1=[]; alpha=[]; K=[]; PHI_0=[]; R2=[]; Erms=[]; return;
end

Report_iterations = 0;
switch lower(CRITERION)
    case 'b'
        w_n = ones(size(C_n));
        [a0,a1,alpha,R2,Erms]=PE1_estimation_bc(phi_n,C_n,w_n,'b',NITER,Report_iterations);
    case 'c'
        cond = C_n>0;
        if sum(cond)~=length(phi_n)
            fprintf('...Warning!: null concentration ignored for (c) criterion.\n');
            phi_n=phi_n(cond); C_n=C_n(cond);
        end
        w_n = 1./(C_n.*C_n);
        [a0,a1,alpha,R2,Erms]=PE1_estimation_bc(phi_n,C_n,w_n,'c',NITER,Report_iterations);
    case 'a'
```

```
        [a0,a1,alpha,R2,Erms]=PE1_estimation_a(phi_n,C_n,NITER,Report_iterations);
    otherwise
        fprintf('Error: CRITERION should be ''a'' ''A'' ''b'' ''B'' ''c'' or ''C''\n');
        a0=[]; a1=[]; alpha=[]; K=[]; PHI_0=[]; R2=[]; Erms=[]; return;
end

PHI_0 = (-a0/a1).^(1/alpha);
K = abs(1/(a0*alpha));
if DIAGRAM
    figure(DIAGRAM)
    D_phi=(max(phi_n)-min(phi_n))/200;
    x_grafica = min(phi_n):D_phi:max(phi_n);
    y=a0+a1*x_grafica.^alpha;
    plot(x_grafica,y,'-r',phi_n,C_n,'ok')
    xlabel('analytical signal')
    ylabel('concentration (%)')
end
if REPORT
    fprintf('Pol.E-1(%s): E=%.4f  R2=%.4f  a0=%.4f  a1=%.4f  alpha=%.4f  PHI_0=%.4f  K=%f\n',...
        CRITERION,Erms,R2,a0,a1,alpha,PHI_0,K);
end
return;
%%%%%%%%%%%%%%%%%%%%%%%%%%%%%%%%%%%%%%%%%%%%%%%%%%%%%%%%%%%%%%%%%%%%%%%%%%
function [a0,a1,alpha,R2,Erms]=PE1_estimation_a(phi_n,C_n,NITER,REPORT)
% pre-scaling
phi_scale=max(phi_n);
phi_n=phi_n/phi_scale;
% iterative estimation of alpha for criterion (a)
% first step: initalization with PE2(c)
w_a=zeros(size(C_n));
cond=C_n>0;
w_a(cond)=1./(C_n(cond).^2);
[a0,a1,alpha]=PE1_estimation_bc(phi_n,C_n,w_a,'c',ceil(NITER/2),0);
% reestimation of weights and new iterations:
for iter=1:5
    f1=phi_n.^(alpha-1);
    w_a=(abs(a1*alpha*f1)).^(-2);
    [a0,a1,alpha]=PE1_estimation_bc(phi_n,C_n,w_a,'c',ceil(NITER/5),0);
    phi_estim = ((C_n-a0)/a1).^(1/alpha);
    E0=mean((phi_estim - phi_n).^2);
    if REPORT, fprintf('Iter:%d  E:%f  a0:%f  a1:%f  alpha:%f\n',iter*ceil(NITER/5),sqrt(E0),a0,a1,alpha); end;
end
% de-scaling
phi_n=phi_n*phi_scale;
a1=a1/phi_scale^alpha;
% statistical results
phi_estim = ((C_n-a0)/a1).^(1/alpha);
SS_res = mean((phi_estim - phi_n).^2);
Erms = sqrt(SS_res);
mu = mean(phi_n);
SS_tot = mean((phi_n - mu).^2);
R2 = 1 - SS_res/SS_tot;
return;
%%%%%%%%%%%%%%%%%%%%%%%%%%%%%%%%%%%%%%%%%%%%%%%%%%%%%%%%%%%%%%%%%%%%%%%%%%
function [a0,a1,alpha,R2,Erms]=PE1_estimation_bc(phi_n,C_n,w_n,CRITERION,NITER,REPORT)
% pre-scaling
phi_scale=max(phi_n);
phi_n=phi_n/phi_scale;
% initialization of alpha:
alpha_search = -1.*10.^(-3:0.02:3); % exhaustive search around -1
alpha_ref=0.0; E0_ref=1e30;
warning('off');
for iter = 1:length(alpha_search)
    [E0] = Error_estimation_PE1_bc(phi_n,C_n,w_n,alpha_search(iter));
    if E0<E0_ref, E0_ref=E0; alpha_ref=alpha_search(iter); end;
    if REPORT, fprintf('Init: alpha=%f   E=%f\n',alpha_search(iter),sqrt(E0)); end;
end
warning('on');
alpha=alpha_ref;
% using alpha as initialization, 1-Dim search using Newton's method:
for iter=1:NITER
    epsilon = abs(alpha/1000);
    x0=alpha; xa=x0-epsilon; xb=x0+epsilon;
    [E0,a0,a1] = Error_estimation_PE1_bc(phi_n,C_n,w_n,x0);
    [Ea] = Error_estimation_PE1_bc(phi_n,C_n,w_n,xa);
```

```
    [Eb] = Error_estimation_PE1_bc(phi_n,C_n,w_n,xb);
    Dx0=-epsilon/2*(Eb-Ea)/(Eb+Ea-2*E0);
    if REPORT, fprintf('Iter:%d  E:%f  a0:%f  a1:%f  alpha:%f\n',iter,sqrt(E0),a0,a1,alpha); end;
    % usually, k=k+Dx0; however Newton's method finds minima or maxima...
    % so, we analyze several options to guarantee minimization
    Dx=[1 0.5 -0.5 -1].*Dx0;
    E0_verify=E0; alpha0=alpha;
    for idx=1:4
        [E_verify] = Error_estimation_PE1_bc(phi_n,C_n,w_n,alpha0+Dx(idx));
        if E_verify<E0_verify
            E0_verify=E_verify;
            alpha=alpha0+Dx(idx);
        end
    end
    if E0_verify==E0
        alpha=alpha0+0.05*Dx0;
    end
end
[E0,a0,a1] = Error_estimation_PE1_bc(phi_n,C_n,w_n,alpha);
% de-scaling
phi_n=phi_n*phi_scale;
a1=a1/phi_scale^alpha;
% statistical results
C_estim=a0+a1*phi_n.^alpha;
SS_res = mean(w_n.*(C_estim - C_n).^2);
Erms = sqrt(SS_res);
mu = mean(C_n);
SS_tot = mean(w_n.*(C_n - mu).^2);
R2 = 1 - SS_res/SS_tot;
if CRITERION == 'c'
    R2=1-SS_res;
end
return;
%%%%%%%%%%%%%%%%%%%%%%%%%%%%%%%%%%%%%%%%%%%%%%%%%%%%%%%%%%%%%%%%%%%%%%%%%%%
function [E,a0,a1] = Error_estimation_PE1_bc(phi_n,C_n,w_n,alpha)
% definitions
f=phi_n.^alpha;
X=ones(size(C_n));
Xf=X.*f; Xff=Xf.*f;
Xc=C_n; Xcf=Xc.*f;
% elements of matrices
A0=sum(w_n.*X); A1=sum(w_n.*Xf); A2=sum(w_n.*Xff);
B0=sum(w_n.*Xc); B1=sum(w_n.*Xcf);
% matrices
A=[A0 A1; A1 A2];
B=[B0; B1];
% coeficients a0 a1 a2:
x=A\B; a0=x(1); a1=x(2);
% error estimation
C_estim = a0 + a1*f;
E=mean(w_n.*(C_estim-C_n).^2);
return;
%%%%%%%%%%%%%%%%%%%%%%%%%%%%%%%%%%%%%%%%%%%%%%%%%%%%%%%%%%%%%%%%%%%%%%%%%%%
```

2.3. Function for Fitting the 2nd Degree Polynomial-Exponent Model: Model_PE2.m

```
%%%%%%%%%%%%%%%%%%%%%%%%%%%%%%%%%%%%%%%%%%%%%%%%%%%%%%%%%%%%%%%%%%%%%%%%%%%
% function [a0,a1,a2,alpha,PHI_0,K,R2,Erms] = model_PE2(phi_n,C_n,NITER,CRITERION,DIAGRAM,REPORT)
%%%%%%%%%%%%%%%%%%%%%%%%%%%%%%%%%%%%%%%%%%%%%%%%%%%%%%%%%%%%%%%%%%%%%%%%%%%%%%%%%%%%
%%% Developed by A. de la Torre, S. Medina-Rodriguez, JC. Segura and
%%%   J.F. Fernandez-Sanchez
%%%   (atv@ugr.es smedina@ugr.es, segura@ugr.es, jffernan@ugr.es)
%%% Dpt. Signal Theory Networking and Communications / Dpt. Analytical Chemistry
%%% University of Granada, May 2020
%%%%%%%%%%%%%%%%%%%%%%%%%%%%%%%%%%%%%%%%%%%%%%%%%%%%%%%%%%%%%%%%%%%%%%%%%%%%%%%%%%%%
%   This function provides an estimation of the parameters of the
% 2nd order Polynomic-Exponent model (PE2) given observations of
% photoluminiscece consisting on analytical signal and concentration (phi_n, C_n)
%   The variable NITER indicates the number of iterations
%   The variable CRITERION defines the calibration criterion ('A' 'B' 'C')
%   The variables DIAGRAM and REPORT provides some information and plots
% while the model parameters are estimated
%   The output parameters are:
%        a0,a1,a2   The polynomial coefficients
%        alpha      The exponent
```

```
%        PHI_0      The response of the model at null concentration
%        K          The sensitivity of the model
%        R2         Determination coefficient
%        Erms       RMS error for calibration data
%   PE2 model:
%        C = a0 + a1 phi^alpha + a2 phi^(2*alpha)
%
%   Procedure for optimization:
%      it is a 1-D search implemented with Newton's method
%
%    Example:
%         C_n=[0.01 0.03 0.1 0.3 1];
%         phi_n=[35 30 25 20 15];
%         [a0,a1,a2,alpha,PHI_0,K,R2,Erms] = model_PE2(phi_n,C_n,100,'c',1,1);
%%%%%%%%%%%%%%%%%%%%%%%%%%%%%%%%%%%%%%%%%%%%%%%%%%%%%%%%%%%%%%%%%%%%%%%%%%
function [a0,a1,a2,alpha,PHI_0,K,R2,Erms] = model_PE2(phi_n,C_n,NITER,CRITERION,DIAGRAM,REPORT)
N=length(C_n);
if N~=length(phi_n)
    fprintf('Error: phi_n and C_n should have the same length\n');
    a0=[]; a1=[]; a2=[]; alpha=[]; K=[]; PHI_0=[]; R2=[]; Erms=[]; return;
end

Report_iterations = 0;
switch lower(CRITERION)
    case 'b'
        w_n = ones(size(C_n));
        [a0,a1,a2,alpha,R2,Erms]=PE2_estimation_bc(phi_n,C_n,w_n,'b',NITER,Report_iterations);
    case 'c'
        cond = C_n>0;
        if sum(cond)~=length(phi_n)
            fprintf('...Warning!: null concentration ignored for (c) criterion.\n');
            phi_n=phi_n(cond); C_n=C_n(cond);
        end
        w_n = 1./(C_n.*C_n);
        [a0,a1,a2,alpha,R2,Erms]=PE2_estimation_bc(phi_n,C_n,w_n,'c',NITER,Report_iterations);
    case 'a'
        [a0,a1,a2,alpha,R2,Erms]=PE2_estimation_a(phi_n,C_n,NITER,Report_iterations);
    otherwise
        fprintf('Error: CRITERION should be ''a'' ''A'' ''b'' ''B'' ''c'' or ''C''\n');
        a0=[]; a1=[]; a2=[]; alpha=[]; K=[]; PHI_0=[]; R2=[]; Erms=[]; return;
end

PHI_0 = ((-a1 + sqrt(a1*a1-4*a0*a2))/(2*a2))^(1/alpha);
K = abs(1/(alpha*a1*PHI_0^alpha+2*alpha*a2*PHI_0^(2*alpha)));
if DIAGRAM
    figure(DIAGRAM)
    D_phi=(max(phi_n)-min(phi_n))/200;
    x_grafica = min(phi_n):D_phi:max(phi_n);
    y=a0+a1*x_grafica.^alpha+a2*x_grafica.^(2*alpha);
    plot(x_grafica,y,'-r',phi_n,C_n,'ok')
    xlabel('analytical signal')
    ylabel('concentration (%)')
end
if REPORT
    fprintf('Pol.E-2(%s): E=%.4f  R2=%.4f  a0=%.4f  a1=%.4f  a2=%.4f  alpha=%.4f  PHI_0=%.4f  K=%f\n',...
        CRITERION,Erms,R2,a0,a1,a2,alpha,PHI_0,K);
end
return;
%%%%%%%%%%%%%%%%%%%%%%%%%%%%%%%%%%%%%%%%%%%%%%%%%%%%%%%%%%%%%%%%%%%%%%%%%%
function [a0,a1,a2,alpha,R2,Erms]=PE2_estimation_a(phi_n,C_n,NITER,REPORT)
% pre-scaling
phi_scale=max(phi_n);
phi_n=phi_n/phi_scale;
% iterative estimation of alpha for criterion (a)
% first step: initalization with PE2(c)
w_a=zeros(size(C_n));
cond=C_n>0;
w_a(cond)=1./(C_n(cond).^2);
[a0,a1,a2,alpha]=PE2_estimation_bc(phi_n,C_n,w_a,'c',ceil(NITER/2),0);
% reestimation of weights and new iterations:
for iter=1:5
    f1=phi_n.^(alpha-1);
    f2=phi_n.^(2*alpha-1);
    w_a=(abs(a1*alpha*f1 + 2*a2*alpha*f2)).^(-2);
    [a0,a1,a2,alpha]=PE2_estimation_bc(phi_n,C_n,w_a,'c',ceil(NITER/5),0);
```

```
    phi_estim = ((-a1+sqrt(a1*a1-4*a2*(a0-C_n)))/(2*a2)).^(1/alpha);
    E0=mean((phi_estim - phi_n).^2);
    if REPORT, fprintf('Iter:%d  E:%f  a0:%f  a1:%f  a2=%f  alpha:%f\n',iter*ceil(NITER/5),sqrt(E0),a0,a1,a2,alpha); end;
end
% de-scaling
phi_n=phi_n*phi_scale;
a1=a1/phi_scale^alpha;
a2=a2/phi_scale^(2*alpha);
% statistical results
phi_estim = ((-a1+sqrt(a1*a1-4*a2*(a0-C_n)))/(2*a2)).^(1/alpha);
SS_res = mean((phi_estim - phi_n).^2);
Erms = sqrt(SS_res);
mu = mean(phi_n);
SS_tot = mean((phi_n - mu).^2);
R2 = 1 - SS_res/SS_tot;
return;
%%%%%%%%%%%%%%%%%%%%%%%%%%%%%%%%%%%%%%%%%%%%%%%%%%%%%%%%%%%%%%%%%%%%%%%%%%%%%%%%%%%%%%
function [a0,a1,a2,alpha,R2,Erms]=PE2_estimation_bc(phi_n,C_n,w_n,CRITERION,NITER,REPORT)
% pre-scaling
phi_scale=max(phi_n);
phi_n=phi_n/phi_scale;
% initialization of alpha:
alpha_search = -1.*10.^(-3:0.02:3); % exhaustive search around -1
alpha_ref=0.0; E0_ref=1e30;
warning('off');
for iter = 1:length(alpha_search)
    [E0] = Error_estimation_PE2_bc(phi_n,C_n,w_n,alpha_search(iter));
    if E0<E0_ref, E0_ref=E0; alpha_ref=alpha_search(iter); end;
    if REPORT, fprintf('Init: alpha=%f   E=%f\n',alpha_search(iter),sqrt(E0)); end;
end
warning('on');
alpha=alpha_ref;
% using alpha as initialization, 1-Dim search using Newton's method:
for iter=1:NITER
    epsilon = abs(alpha/1000);
    x0=alpha; xa=x0-epsilon; xb=x0+epsilon;
    [E0,a0,a1,a2] = Error_estimation_PE2_bc(phi_n,C_n,w_n,x0);
    [Ea] = Error_estimation_PE2_bc(phi_n,C_n,w_n,xa);
    [Eb] = Error_estimation_PE2_bc(phi_n,C_n,w_n,xb);
    Dx0=-epsilon/2*(Eb-Ea)/(Eb+Ea-2*E0);
    if REPORT, fprintf('Iter:%d  E:%f  a0:%f  a1:%f  a2=%f  alpha:%f\n',iter,sqrt(E0),a0,a1,a2,alpha); end;
    % usually, k=k+Dx0; however Newton's method finds minima or maxima...
    % so, we analyze several options to guarantee minimization
    Dx=[1 0.5 -0.5 -1].*Dx0;
    E0_verify=E0; alpha0=alpha;
    for idx=1:4
        [E_verify] = Error_estimation_PE2_bc(phi_n,C_n,w_n,alpha0+Dx(idx));
        if E_verify<E0_verify
            E0_verify=E_verify;
            alpha=alpha0+Dx(idx);
        end
    end
    if E0_verify==E0
        alpha=alpha0+0.05*Dx0;
    end
end
[E0,a0,a1,a2] = Error_estimation_PE2_bc(phi_n,C_n,w_n,alpha);
% de-scaling
phi_n=phi_n*phi_scale;
a1=a1/phi_scale^alpha;
a2=a2/phi_scale^(2*alpha);
% statistical results
C_estim=a0+a1*phi_n.^alpha+a2*phi_n.^(2*alpha);
SS_res = mean(w_n.*(C_estim - C_n).^2);
Erms = sqrt(SS_res);
mu = mean(C_n);
SS_tot = mean(w_n.*(C_n - mu).^2);
R2 = 1 - SS_res/SS_tot;
if CRITERION == 'c'
    R2=1-SS_res;
end
return;
%%%%%%%%%%%%%%%%%%%%%%%%%%%%%%%%%%%%%%%%%%%%%%%%%%%%%%%%%%%%%%%%%%%%%%%%%%%%%%%%%%%%%%
function [E,a0,a1,a2] = Error_estimation_PE2_bc(phi_n,C_n,w_n,alpha)
% definitions
```

```
f=phi_n.^alpha;
X=ones(size(C_n));
Xf=X.*f; Xff=Xf.*f; Xfff=Xff.*f; Xffff=Xfff.*f;
Xc=C_n; Xcf=Xc.*f; Xcff=Xcf.*f;
% elements of matrices
A0=sum(w_n.*X); A1=sum(w_n.*Xf); A2=sum(w_n.*Xff);
A3=sum(w_n.*Xfff); A4=sum(w_n.*Xffff);
B0=sum(w_n.*Xc); B1=sum(w_n.*Xcf); B2=sum(w_n.*Xcff);
% matrices
A=[A0 A1 A2; A1 A2 A3; A2 A3 A4];
B=[B0; B1; B2];
% coeficients a0 a1 a2:
x=A\B; a0=x(1); a1=x(2); a2=x(3);
% error estimation
C_estim = a0 + a1*f + a2*f.*f;
E=mean(w_n.*(C_estim-C_n).^2);
return;
%%%%%%%%%%%%%%%%%%%%%%%%%%%%%%%%%%%%%%%%%%%%%%%%%%%%%%%%%%%%%%%%%%%%%%%%%%%
```

2.4. *An Example Script Using the Calibration Functions: Script_Calibration_P2_PE1_PE2.m*

```
%%%%%%%%%%%%%%%%%%%%%%%%%%%%%%%%%%%%%%%%%%%%%%%%%%%%%%%%%%%%%%%%%%%%%%%%%%%
% script_calibration_P2_PE1_PE2.m
%%%%%%%%%%%%%%%%%%%%%%%%%%%%%%%%%%%%%%%%%%%%%%%%%%%%%%%%%%%%%%%%%%%%%%%%%%%%%%%%%%%%%%
clear; clc;

% Calibration data:
% 1st column: pO2 (kPa); 2nd column: phase-shift mean (deg); 3rd column std-dev
A=[   1.00 53.4867 1.3362
      2.00 46.4084 1.2695
      3.00 41.8561 1.2056
      4.00 38.0862 1.5058
      5.00 34.6263 1.2201
      6.00 32.8769 1.5728
      7.00 30.1863 1.3458
      8.00 28.8031 1.5315
      9.00 26.8341 1.7249
      10.00 25.9402 1.3633
      12.00 23.5732 1.9227
      14.00 21.3130 1.6939
      16.00 19.5109 1.9817
      18.00 17.7437 2.2541
      20.00 16.6199 2.2002 ];
C_n=A(:,1);
phi_n=A(:,2);
CRITERION='c';

%% variable phi for plots
Dphi=(max(phi_n)-min(phi_n))/200;
phi=(min(phi_n):Dphi:max(phi_n))';
Range_x = [ min(phi)-10*Dphi max(phi)+10*Dphi ];

%% P2 calibration (model polynomial-2nd degree)
NITER=1;
[a0,a1,a2,PHI_0,K,R2,Erms] = model_P2(phi_n,C_n,NITER,CRITERION,0,0);
% Reporting calibration
fprintf('POLINOMIAL-2nd DEGREE calibration\n');
fprintf('Parameter\n');
fprintf('a_0:      %10.6f\n',a0);
fprintf('a_1:      %10.6f\n',a1);
fprintf('a_2:      %10.6f\n',a2);
fprintf('R2:       %10.8f\n',R2);
fprintf('RMSE:     %10.6f %%\n',Erms*100);
fprintf('Iter.:    %10d\n',NITER);
fprintf('---------------------------------------------\n');
fprintf('Phi_0:    %10.6f\n',PHI_0);
fprintf('K:        %10.6f\n',K);
fprintf('=============================================\n');
% plot with calibration curves
C_P2=a0+a1./phi+a2./phi./phi;

%% PE1 calibration: model polynomial-exponent 1st degree
NITER=1;
[a0,a1,alpha,PHI_0,K,R2,Erms] = model_PE1(phi_n,C_n,NITER,CRITERION,0,0);
% Reporting calibration
```

```
fprintf('POLINOMIAL-EXPONENT-1st DEGREE calibration\n');
fprintf('Parameter\n');
fprintf('a_0:      %10.6f\n',a0);
fprintf('a_1:      %10.6f\n',a1);
fprintf('alpha:    %10.6f\n',alpha);
fprintf('R2:       %10.8f\n',R2);
fprintf('RMSE:     %10.6f %%\n',Erms*100);
fprintf('Iter.:    %10d\n',NITER);
fprintf('---------------------------------------------\n');
fprintf('Phi_0:    %10.6f\n',PHI_0);
fprintf('K:        %10.6f\n',K);
fprintf('=============================================\n');
% plot with calibration curves
C_P1E=a0+a1.*phi.^(alpha);

%% PE2 calibration: model polynomial-exponent 2nd degree
[a0,a1,a2,alpha,PHI_0,K,R2,Erms] = model_PE2(phi_n,C_n,NITER,CRITERION,0,0);
% Reporting calibration
fprintf('POLINOMIAL-EXPONENT-2nd DEGREE calibration\n');
fprintf('Parameter\n');
fprintf('a_0:      %10.6f\n',a0);
fprintf('a_1:      %10.6f\n',a1);
fprintf('a_2:      %10.6f\n',a2);
fprintf('alpha:    %10.6f\n',alpha);
fprintf('R2:       %10.8f\n',R2);
fprintf('RMSE:     %10.6f %%\n',Erms*100);
fprintf('Iter.:    %10d\n',100);
fprintf('---------------------------------------------\n');
fprintf('Phi_0:    %10.6f\n',PHI_0);
fprintf('K:        %10.6f\n',K);
fprintf('=============================================\n');
% plot with calibration curves
C_P2E=a0+a1.*(phi.^(alpha))+a2.*(phi.^(2*alpha));

% calibration figurefigure(1)
p_fig=plot(phi,[C_P2 C_P1E C_P2E],phi_n,C_n,'ok');
set(p_fig(4),'MarkerSize',6,'MarkerFaceColor','k');
xlim(Range_x); grid on;
xlabel('analytical signal: phase-shift (degrees)');
ylabel('concentration: pO_2 (kPa)');
legend('Polyn-2','Polyn-Exp-1','Polyn-Exp-2','data');

return;
%%%%%
```

3. Statistics of the Calibration and Evaluation Data-Sets for Each Experiment

Experiment 1: PtTFPP/PS sensor using ϕ
Analytical signal: phase-shift, ϕ (^{o}) as provided by the instrument

	Calibration data set (N=125 meas./pO_2)		Evaluation data set (N=125 meas./pO_2)	
pO_2 (kPa)	Mean (^{o})	Std.Dev. (^{o})	Mean (^{o})	Std.Dev. (^{o})
0.50	61.1859	0.3723	61.1918	0.4012
0.75	59.6414	0.3529	59.5448	0.3256
1.00	58.0924	0.4532	58.0448	0.3588
2.00	52.6697	0.4230	52.7366	0.3902
3.00	48.1880	0.3818	48.2234	0.3321
4.00	44.5679	0.3485	44.5732	0.4112
5.00	41.5163	0.4405	41.4578	0.3793
6.00	38.7990	0.3474	38.8548	0.3650
7.00	36.6220	0.3660	36.5494	0.3794
8.00	34.5674	0.4020	34.5973	0.4610
9.00	32.8415	0.4919	32.8300	0.4354
10.00	31.1980	0.4564	31.2210	0.4339
12.00	28.3866	0.4351	28.4430	0.4712
14.00	26.0140	0.5563	26.0257	0.4743
16.00	23.8549	0.6641	23.8962	0.6078
18.00	21.7150	0.6193	21.7474	0.6393
20.00	20.2151	0.6376	20.3674	0.5465

Experiment 2: PtTFPP/PS sensor using τ_ϕ
Analytical signal: ϕ-based lifetime, τ_ϕ (μs), $\tau_\phi = -\tan(\phi)/(2\pi f)$
(see eq. (9) in manuscript).

	Calibration data set (N=125 meas./pO_2)		Evaluation data set (N=125 meas./pO_2)	
pO_2 (kPa)	Mean (μs)	Std.Dev. (μs)	Mean (μs)	Std.Dev. (μs)
0.50	56.2460	0.8686	56.2614	0.9413
0.75	52.8208	0.7515	52.6160	0.6853
1.00	49.6938	0.8767	49.5977	0.6892
2.00	40.5679	0.6201	40.6655	0.5730
3.00	34.5866	0.4621	34.6287	0.4034
4.00	30.4731	0.3713	30.4794	0.4388
5.00	27.3866	0.4243	27.3295	0.3661
6.00	24.8721	0.3083	24.9219	0.3255
7.00	22.9934	0.3069	22.9327	0.3173
8.00	21.3155	0.3192	21.3397	0.3672
9.00	19.9694	0.3759	19.9601	0.3322
10.00	18.7343	0.3367	18.7512	0.3205
12.00	16.7177	0.3039	16.7574	0.3296
14.00	15.0986	0.3719	15.1060	0.3176
16.00	13.6810	0.4292	13.7074	0.3934
18.00	12.3211	0.3862	12.3415	0.4004
20.00	11.3923	0.3910	11.4853	0.3361

**Experiment 3: PtTFPP/PS sensor using m**<br>Analytical signal: modulation factor, m (mV/V) as provided by the instrument				
	Calibration data set (N=125 meas./pO_2)		Evaluation data set (N=125 meas./pO_2)	
pO_2 (kPa)	Mean (mV/V)	Std.Dev. (mV/V)	Mean (mV/V)	Std.Dev. (mV/V)
0.50	0.7339	0.0047	0.7348	0.0051
0.75	0.7235	0.0057	0.7229	0.0052
1.00	0.7103	0.0041	0.7121	0.0045
2.00	0.6586	0.0039	0.6580	0.0046
3.00	0.6077	0.0038	0.6078	0.0037
4.00	0.5619	0.0046	0.5606	0.0039
5.00	0.5227	0.0032	0.5219	0.0034
6.00	0.4871	0.0033	0.4872	0.0031
7.00	0.4558	0.0036	0.4559	0.0031
8.00	0.4295	0.0038	0.4299	0.0031
9.00	0.4060	0.0033	0.4061	0.0035
10.00	0.3842	0.0031	0.3848	0.0031
12.00	0.3479	0.0031	0.3480	0.0031
14.00	0.3171	0.0030	0.3178	0.0033
16.00	0.2913	0.0025	0.2918	0.0028
18.00	0.2680	0.0029	0.2681	0.0027
20.00	0.2515	0.0026	0.2513	0.0027

**Experiment 4: PtTFPP/PS sensor using τ_m**<br>Analytical signal: m-based lifetime, τ_m (μs) (according to eq. (11) in manuscript, with $\tau_0 = 64.7231$ μs, m_0=0.756667 mV/V)				
	Calibration data set (N=125 meas./pO_2)		Evaluation data set (N=125 meas./pO_2)	
pO_2 (kPa)	Mean (μs)	Std.Dev. (μs)	Mean (μs)	Std.Dev. (μs)
0.50	55.9921	1.5254	56.2768	1.7141
0.75	52.8101	1.6108	52.6503	1.4846
1.00	49.2964	1.0119	49.7459	1.1242
2.00	39.2537	0.6119	39.1610	0.7072
3.00	32.5346	0.4282	32.5446	0.4182
4.00	27.9194	0.4158	27.8075	0.3469
5.00	24.6522	0.2449	24.5960	0.2596
6.00	22.0735	0.2230	22.0781	0.2099
7.00	20.0280	0.2256	20.0382	0.1908
8.00	18.4432	0.2202	18.4674	0.1833
9.00	17.1172	0.1832	17.1223	0.1927
10.00	15.9429	0.1640	15.9723	0.1611
12.00	14.1020	0.1520	14.1062	0.1503
14.00	12.6325	0.1383	12.6690	0.1512
16.00	11.4572	0.1117	11.4808	0.1262
18.00	10.4304	0.1257	10.4353	0.1178
20.00	9.7252	0.1107	9.7165	0.1133

**Experiment 5: N1008-AP200/19 sensor using ϕ**				
Analytical signal: phase-shift, ϕ (^{o}) as provided by the instrument				
	Calibration data set (N=50 meas./pO_2)		Evaluation data set (N=50 meas./pO_2)	
pO_2 (kPa)	Mean (^{o})	Std.Dev. (^{o})	Mean (^{o})	Std.Dev. (^{o})
0.25	52.64562	0.04933	52.63426	0.04386
0.50	50.01290	0.01454	50.01720	0.01732
0.75	47.88158	0.01058	47.88426	0.01119
1.00	46.32524	0.00814	46.32666	0.01157
2.00	42.53436	0.00613	42.53372	0.00586
3.00	40.12142	0.00374	40.12062	0.00414
4.00	38.19882	0.00508	38.19920	0.00500
5.00	36.97066	0.00507	36.96934	0.00484
6.00	35.89820	0.00483	35.89776	0.00521
7.00	34.99288	0.00633	34.99116	0.00565
8.00	34.19472	0.00455	34.19730	0.00512
9.00	33.47422	0.00365	33.47376	0.00438
10.00	33.12362	0.00408	33.12418	0.00383
15.00	30.62856	0.00406	30.62938	0.00391
20.00	28.85258	0.00552	28.85154	0.00433

**Experiment 6: N1008-AP200/19 sensor using τ_ϕ**				
Analytical signal: ϕ-based lifetime, τ_ϕ (μs), $\tau_\phi = -\tan(\phi)/(2\pi f)$ (see eq. (9) in manuscript).				
	Calibration data set (N=50 meas./pO_2)		Evaluation data set (N=50 meas./pO_2)	
pO_2 (kPa)	Mean (μs)	Std.Dev. (μs)	Mean (μs)	Std.Dev. (μs)
0.25	6.92726	0.01236	6.92440	0.01100
0.50	6.30431	0.00325	6.30530	0.00387
0.75	5.84805	0.00219	5.84861	0.00230
1.00	5.53798	0.00157	5.53825	0.00225
2.00	4.85097	0.00105	4.85086	0.00100
3.00	4.45590	0.00060	4.45579	0.00066
4.00	4.16071	0.00075	4.16077	0.00073
5.00	3.98020	0.00074	3.98001	0.00069
6.00	3.82729	0.00068	3.82722	0.00073
7.00	3.70139	0.00088	3.70116	0.00078
8.00	3.59269	0.00062	3.59304	0.00070
9.00	3.49632	0.00049	3.49627	0.00058
10.00	3.45002	0.00055	3.45009	0.00051
15.00	3.13060	0.00050	3.13071	0.00049
20.00	2.91317	0.00066	2.91303	0.00052

4. Detailed Experimental Results

4.1. Experiment 1: PtTFPP/PS Sensor Using ϕ

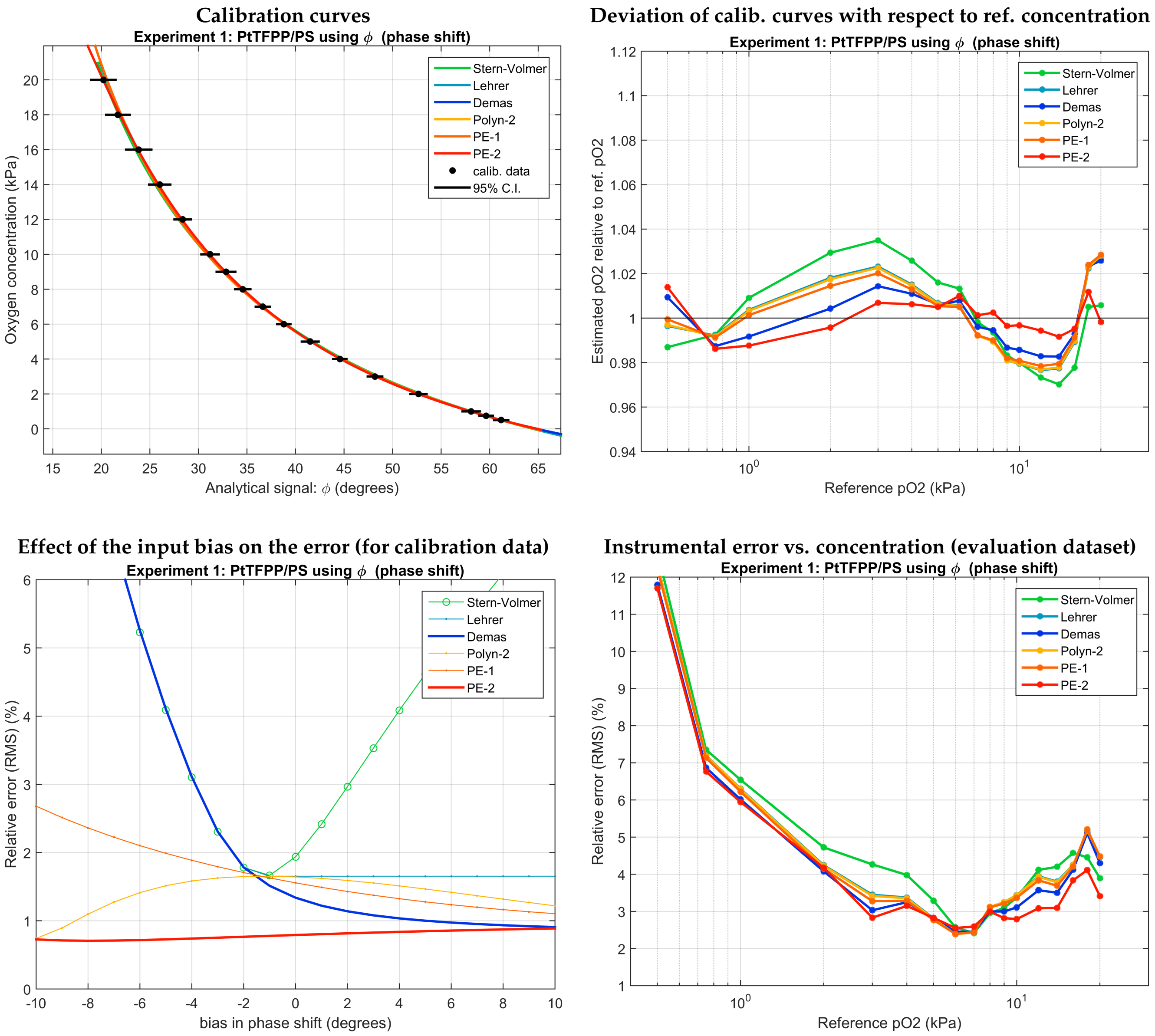

**Experiment 1: PtTFPP/PS sensor (optical fibre) using ϕ**						
Model:	**Stern-Volmer**	**Lehrer**	**Polyn-2**	**Polyn-Expon-1**	**Demas**	**Polyn-Exp-2**
Parameters:	ϕ_0: 64.472	ϕ_0: 64.584	a_0: -8.757	a_0: -8.210	ϕ_0: 64.854	a_0: -5.677
value (std.err)	(0.066)	(0.078)	(0.192)	(0.330)	(0.263)	(0.250)
	k: 0.1088	k:0.1138	a_1: 553.3	a_1: 738.1	k_1: 0.302	a_1: 3.496e3
	(7.4e-4)	(2.3e-3)	(16.9)	(59.3)	(0.389)	(5.83e2)
		x: 0.9808	a_2: 795.9	α: -1.079	k_2: 0.097	a_2: -9.510e4
		(8.1e-3)	(339.9)	(0.028)	(0.012)	(3.75e4)
					x: 0.115	α: -1.528
					(0.181)	(0.049)
Num. param.	2	3	3	3	4	4
Num. iterations	1	100	1	100	500	100
R^2	0.99962457	0.99972677	0.99973022	0.99975794	0.99982092	0.99993740
RMS rel. error (%)	1.937594	1.652978	1.642511	1.555826	1.338195	0.791195
Resp. at $C = 0$ (^{o})	64.472	64.584	64.590	64.629	64.854	64.904
Sens. K_0 (kPa^{-1})	0.1088	0.1116	0.1118	0.1129	0.1207	0.1212
Sens. K_1 (kPa^{-1})	0.0982	0.1000	0.1001	0.1006	0.1027	0.1041
Sens. K_{10} (kPa^{-1})	0.0521	0.0511	0.0510	0.0509	0.0508	0.0500

4.2. Experiment 2: PtTFPP/PS Sensor Using τ_ϕ

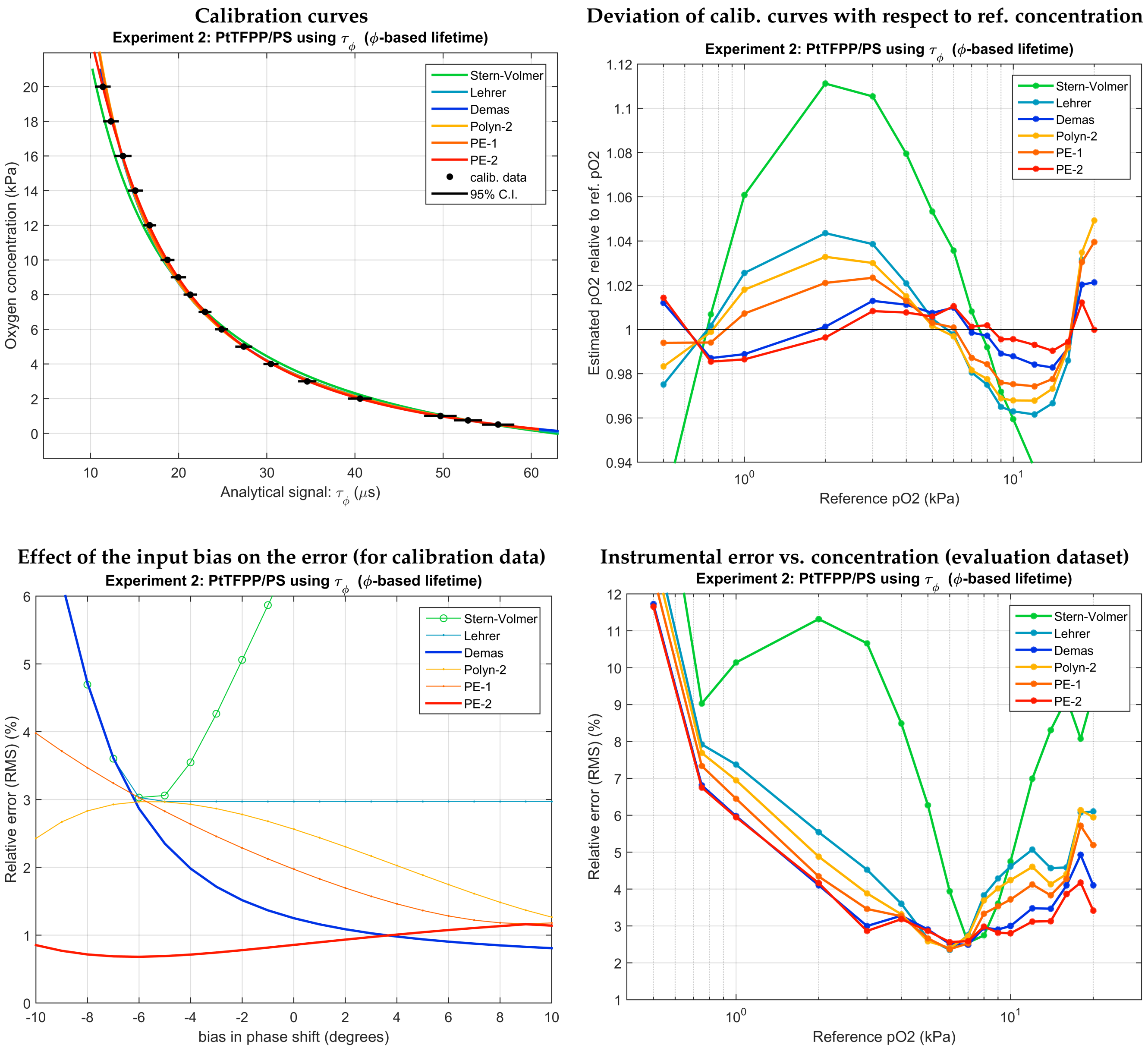

**Experiment 2: PtTFPP/PS sensor (optical fibre) using τ_ϕ**						
Model:	**Stern-Volmer**	**Lehrer**	**Polyn-2**	**Polyn-Expon-1**	**Demas**	**Polyn-Exp-2**
Parameters: value (std.err)	ϕ_0: 62.535 (0.493)<br>k: 0.2437 (6.5e-3)	ϕ_0: 64.003 (0.352)<br>k:0.2991 (9.0e-3)<br>x: 0.9529 (5.1e-3)	a_0: -3.139 (0.116)<br>a_1: 186.3 (8.1)<br>a_2: 1008.9 (112.2)	a_0: -2.431 (0.108)<br>a_1: 544.6 (34.5)<br>α: -1.297 (0.024)	ϕ_0: 66.049 (0.545)<br>k_1: 0.480 (0.064)<br>k_2: 0.091 (0.016)<br>x: 0.689 (0.071)	a_0: -1.751 (0.070)<br>a_1: 1.398e3 (1.32e2)<br>a_2: -1.709e4 (4.28e3)<br>α: -1.590 (0.031)
Num. param.	2	3	3	3	4	4
Num. iterations	1	100	1	100	500	100
R^2	0.99556425	0.99911802	0.99934464	0.99961171	0.99984350	0.99992624
RMS rel. error (%)	6.660145	2.969810	2.560000	1.970503	1.251006	0.858824
Resp. at $C = 0$ (μs)	62.535	64.003	64.336	64.921	66.049	66.197
Sens. K_0 (kPa^{-1})	0.2437	0.2850	0.2956	0.3172	0.3594	0.3649
Sens. K_1 (kPa^{-1})	0.1960	0.2164	0.2201	0.2248	0.2332	0.2346
Sens. K_{10} (kPa^{-1})	0.0709	0.0626	0.0620	0.0620	0.0625	0.0616

4.3. Experiment 3: PtTFPP/PS Sensor Using *m*

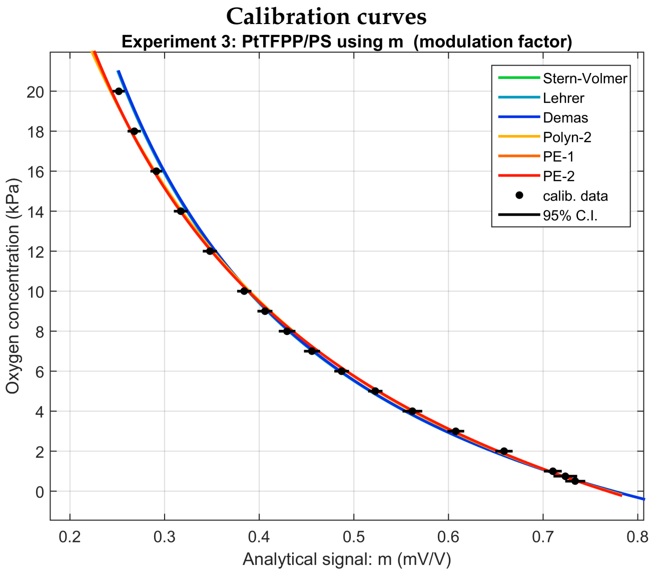

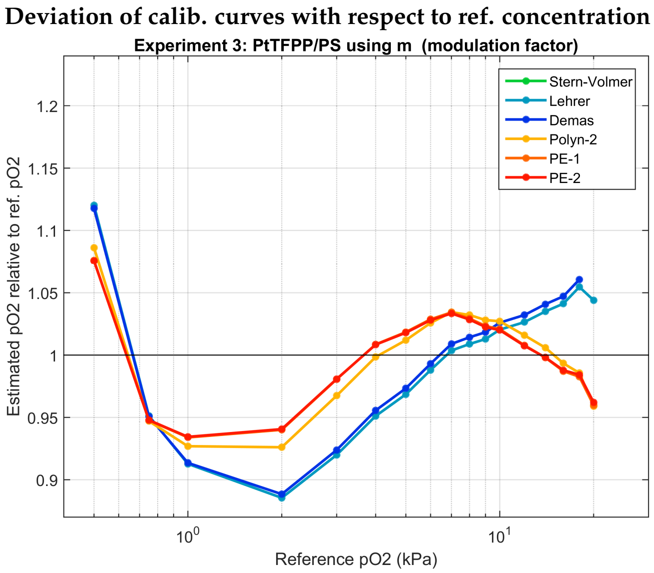

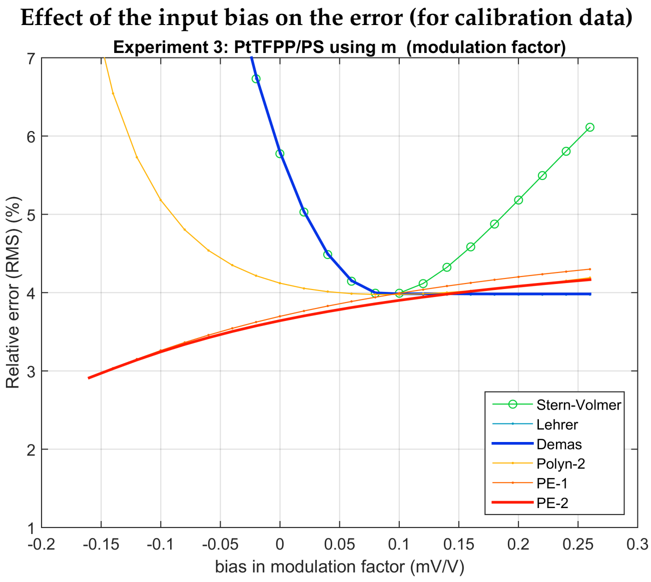

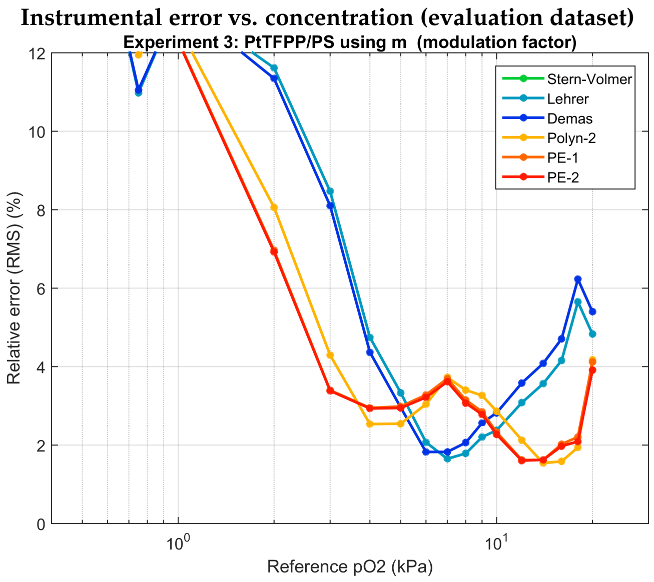

**Experiment 3: PtTFPP/PS sensor (optical fibre) using m**						
Model:	**Stern-Volmer**	**Lehrer**	**Polyn-2**	**Polyn-Expon-1**	**Demas**	**Polyn-Exp-2**
Parameters: value (std.err)	ϕ_0: 0.7749 (2.1e-3)	ϕ_0: 0.7749 (1.9e-3)	a_0: -11.98 (0.55)	a_0: -17.49 (2.53)	ϕ_0: 0.7745 (1.94e-3)	a_0: -19.98 (614.4)
	k: 0.0997 (2.0e-3)	k:0.0997 (2.0e-3)	a_1: 9.897 (0.587)	a_1: 14.68 (2.40)	k_1: 0.0994 (0.109)	a_1: 14.61 (1.12e3)
		x: 1.0000 (0.036)	a_2: -0.5180 (0.141)	α: -0.663 (0.075)	k_2: 0.0990 (1.77e-3)	a_2: -2.546 (1.73e3)
					x: 1.00e-5 (1.53e-2)	α: -0.499 (59.2)
Num. param.	2	3	3	3	4	4
Num. iterations	1	100	1	100	500	100
R^2	0.99666592	0.99666592	0.99830220	0.99863289	0.99664280	0.99867369
RMS rel. error (%)	5.774148	5.774148	4.120435	3.697443	5.794131	3.641851
Resp. at $C = 0$ (mV/V)	0.7749	0.7749	0.7699	0.7682	0.7745	0.7681
Sens. K_0 (kPa^{-1})	0.0997	0.0997	0.0900	0.0862	0.0990	0.0859
Sens. K_1 (kPa^{-1})	0.0906	0.0906	0.0838	0.0815	0.0901	0.0814
Sens. K_{10} (kPa^{-1})	0.0499	0.0499	0.0538	0.0549	0.0498	0.0548

4.4. Experiment 4: PtTFPP/PS Sensor Using τ_m

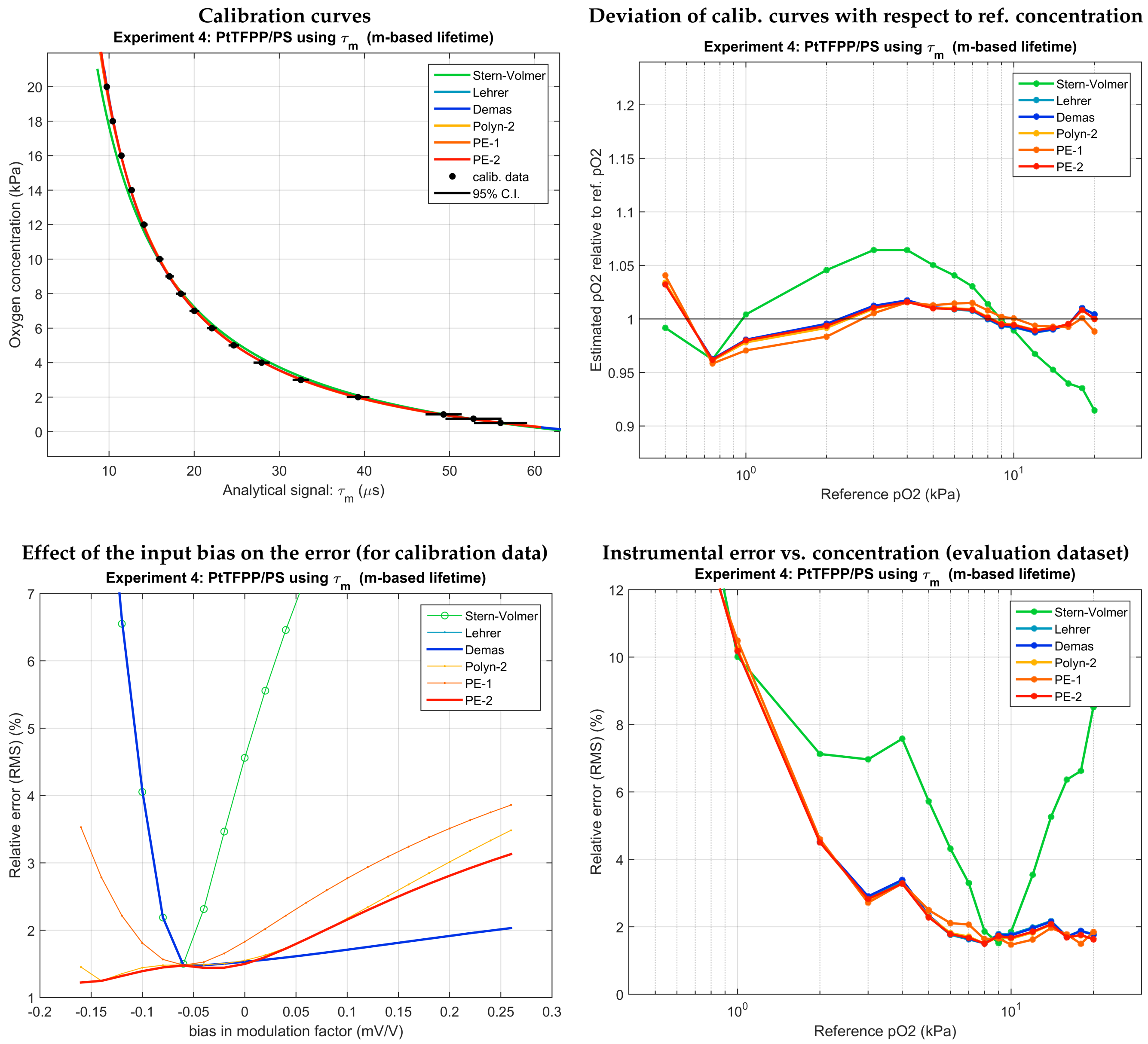

**Experiment 4: PtTFPP/PS sensor (optical fibre) using τ_m**						
Model:	**Stern-Volmer**	**Lehrer**	**Polyn-2**	**Polyn-Expon-1**	**Demas**	**Polyn-Exp-2**
Parameters:<br>value (std.err)	ϕ_0: 64.547 (0.443)<br>k: 0.3082 (6.0e-3)	ϕ_0: 66.063 (0.239)<br>k:0.3613 (5.9e-3)<br>x: 0.9703 (2.3e-3)	a_0: -2.705 (0.057)<br>a_1: 171.9 (3.8)<br>a_2: 475.0 (46.0)	a_0: -2.243 (0.098)<br>a_1: 326.3 (16.9)<br>α: -1.185 (0.021)	ϕ_0: 66.053 (0.367)<br>k_1: 0.361 (0.015)<br>k_2: 3.94e-4 (0.0172)<br>x: 0.970 (0.015)	a_0: -2.989 (0.885)<br>a_1: 94.4 (324.0)<br>a_2: 462.1 (312.4)<br>α: -0.854 (0.724)
Num. param.	2	3	3	3	4	4
Num. iterations	1	100	1	100	500	100
R^2	0.99789800	0.99976111	0.99975561	0.99966228	0.99976105	0.99977127
RMS rel. error (%)	4.584757	1.545611	1.563292	1.837728	1.545814	1.512387
Resp. at $C = 0$ (μs)	64.547	66.063	66.208	66.767	66.053	66.057
Sens. K_0 (kPa^{-1})	0.3082	0.3506	0.3554	0.3761	0.3503	0.3496
Sens. K_1 (kPa^{-1})	0.2357	0.2350	0.2563	0.2602	0.2548	0.2553
Sens. K_{10} (kPa^{-1})	0.0755	0.0686	0.0685	0.0689	0.0687	0.0684

4.5. Experiment 5: N1008-AP200/19 Sensor Using ϕ

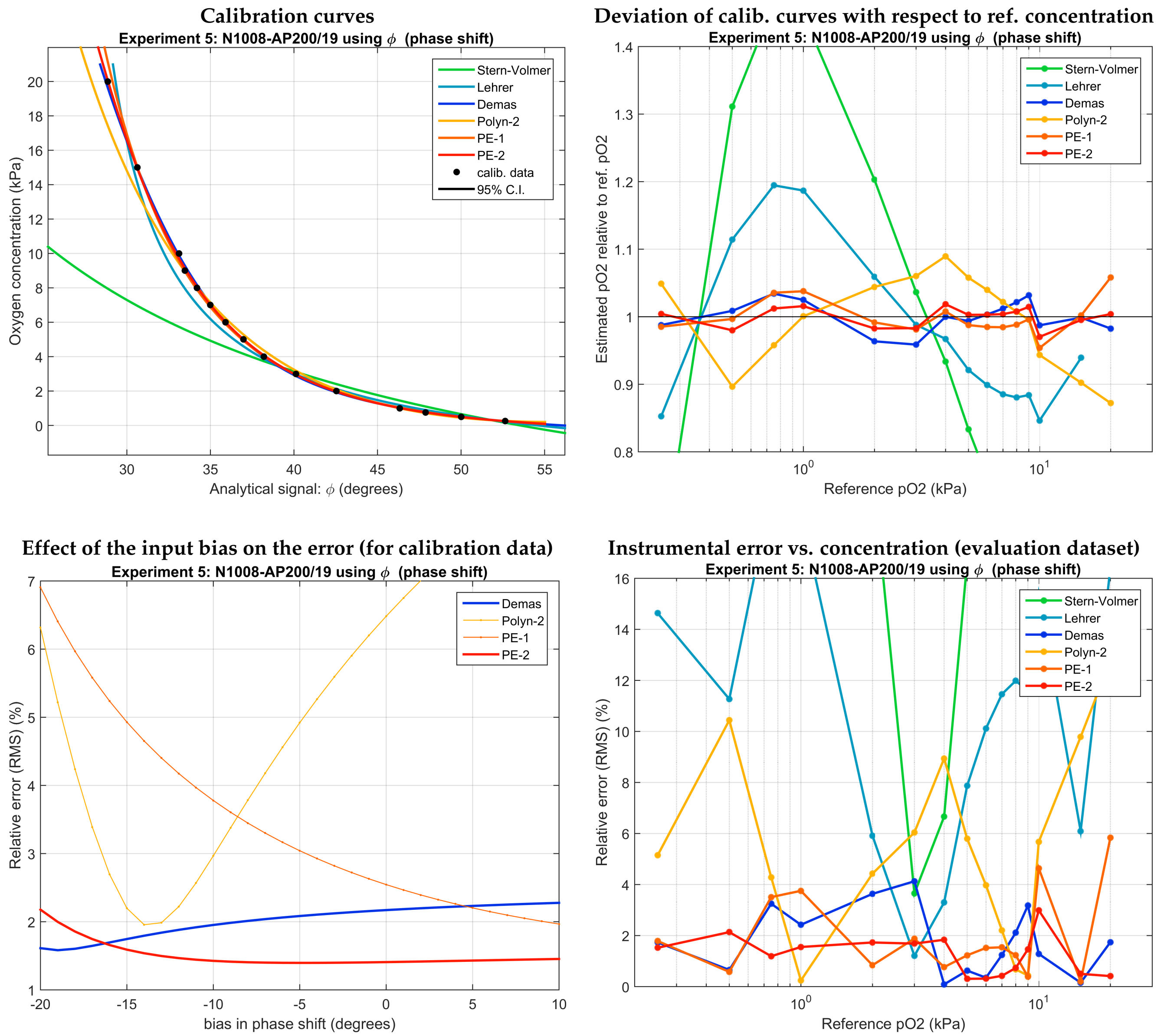

**Experiment 5: N1008-AP200/19 sensor (nanostructured support) using ϕ**						
Model:	**Stern-Volmer**	**Lehrer**	**Polyn-2**	**Polyn-Expon-1**	**Demas**	**Polyn-Exp-2**
Parameters: value (std.err)	ϕ_0: 53.539 (0.576)<br>k: 0.1076 (15.1e-3)	ϕ_0: 54.572 (0.374)<br>k:0.3346 (29.6e-3)<br>x: 0.5318 (8.7e-3)	a_0: 20.31 (1.58)<br>a_1: -2.235e3 (1.44e2)<br>a_2: 6.21e4 (3.2e3)	a_0: -0.516 (0.031)<br>a_1: 2.907e9 (8.2e8)<br>α: -5.566 (0.081)	ϕ_0: 56.069 (0.184)<br>k_1: 0.728 (0.041)<br>k_2: 1.274e-2 (8.7e-4)<br>x: 0.3883 (6.8e-3)	a_0: -0.4056 (2.41e-2)<br>a_1: 1.969e10 (7.11e9)<br>a_2: -3.042e18 (2.54e17)<br>α: -6.085 (0.099)
Num. param.	2	3	3	3	4	4
Num. iterations	1	100	1	100	500	100
R^2	0.86910048	0.98507301	0.99579807	0.99935242	0.99952878	0.99980184
RMS rel. error (%)	36.180039	12.217606	6.482232	2.544760	2.170762	1.407686
Resp. at $C = 0$ (^{o})	53.539	54.572	55.018	56.467	56.069	57.002
Sens. K_0 (kPa^{-1})	0.1076	0.1780	0.2479	0.3480	0.2906	0.4065
Sens. K_1 (kPa^{-1})	0.0971	0.1153	0.1040	0.1185	0.1235	0.1183
Sens. K_{10} (kPa^{-1})	0.0518	0.0160	0.0210	0.0171	0.0174	0.0175

4.6. Experiment 6: N1008-AP200/19 Sensor Using τ_ϕ

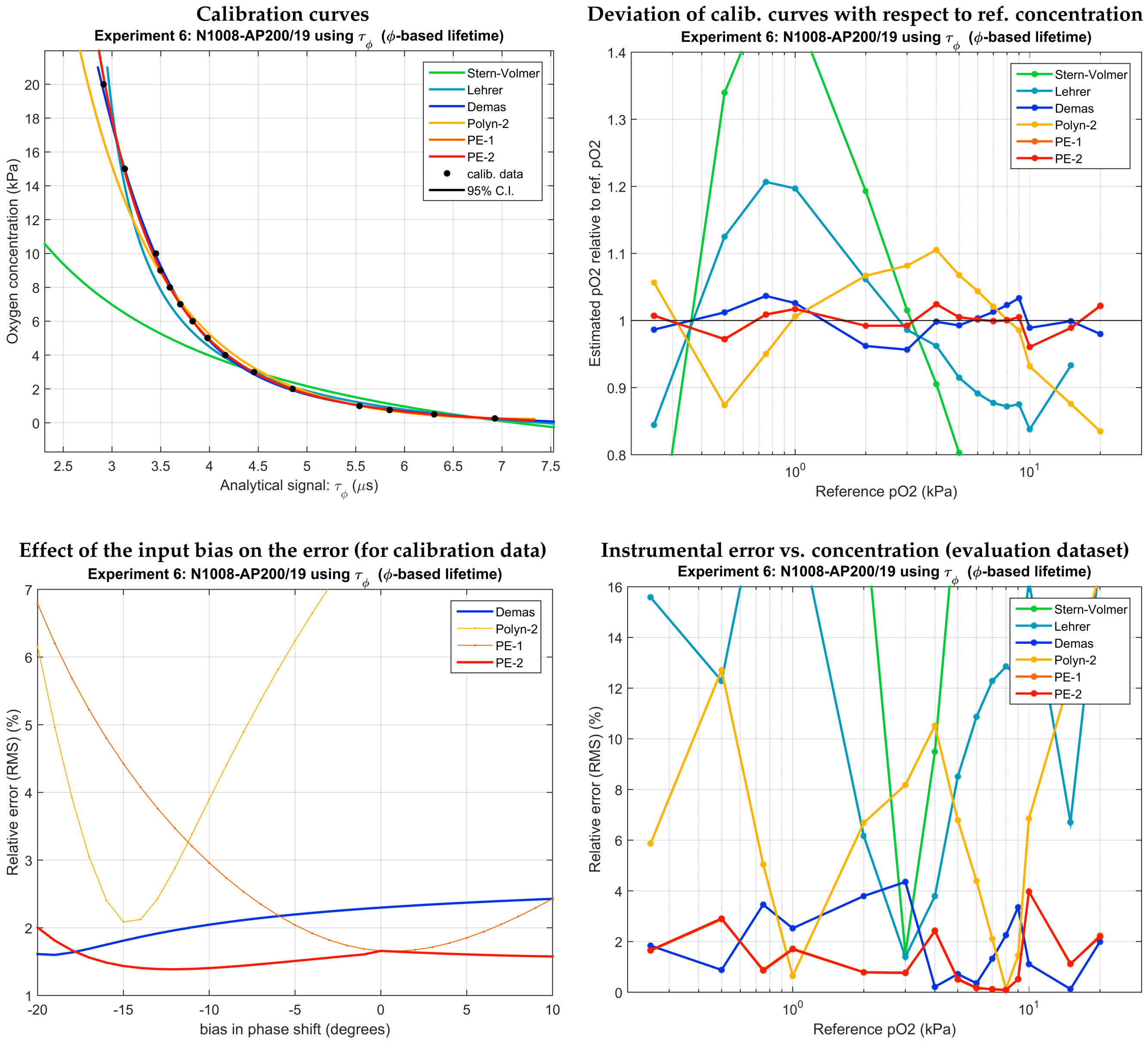

Experiment 6: N1008-AP200/19 sensor (nanostructured support) using τ_ϕ						
Model:	**Stern-Volmer**	**Lehrer**	**Polyn-2**	**Polyn-Expon-1**	**Demas**	**Polyn-Exp-2**
Parameters: value (std.err)	ϕ_0: 7.139 (0.151) k: 0.1977 (3.13e-2)	ϕ_0: 7.408 (0.104) k:0.5224 (4.89e-2) x: 0.6563 (7.3e-3)	a_0: 7.257 (0.784) a_1: -103.6 (8.7) a_2: 381.8 (23.7)	a_0: -0.2044 (0.0139) a_1: 2.284e3 (1.12e2) α: -4.401 (0.038)	ϕ_0: 7.838 (0.056) k_1: 1.081 (0.061) k_2: 1.59e-2 (1.19e-3) x: 0.5442 (5.7e-3)	a_0: -0.1996 (9.24e-2) a_1: -0.700 (13.2) a_2: 2.264e3 (3.89e2) α: -2.195 (0.101)
Num. param.	2	3	3	3	4	4
Num. iterations	1	100	1	100	500	100
R^2	0.85148597	0.98311467	0.99343325	0.99972465	0.99947166	0.99972472
RMS rel. error (%)	38.537517	12.994358	8.103550	1.659374	2.298561	1.659154
Resp. at $C = 0$ (μs)	7.139	7.408	7.134	8.313	7.838	8.321
Sens. K_0 (kPa^{-1})	0.1977	0.3429	0.3828	1.1115	0.5957	1.1220
Sens. K_1 (kPa^{-1})	0.1651	0.1910	0.1624	0.1888	0.2014	0.1888
Sens. K_{10} (kPa^{-1})	0.0664	0.0197	0.0277	0.0223	0.0219	0.0223

© 2020 by the authors. Licensee MDPI, Basel, Switzerland. This article is an open access article distributed under the terms and conditions of the Creative Commons Attribution (CC BY) license (http://creativecommons.org/licenses/by/4.0/).
